# Development of Recombinant Follicle-Stimulating Hormone for the Superovulation of Cattle: A Review

**DOI:** 10.3390/vetsci12030264

**Published:** 2025-03-12

**Authors:** Jiawei Zhang, Haoshu Luo

**Affiliations:** 1 State Key Laboratory of Animal Biotech Breeding, College of Biological Sciences, China Agricultural University, Beijing 100193, China; 2 Beijing VJTBio Co., Ltd., Beijing 100085, China

**Keywords:** superovulation, recombinant protein fusion technology, reproductive hormone, FSH, cattle

## Abstract

Embryo transfer technology is extensively utilized in cattle breeding, with superovulation serving as its foundation. Superovulation currently relies on the administration of exogenous hormones to stimulate follicular development and maturation in the ovaries, with porcine pituitary-derived follicle-stimulating hormone (FSH) being the most commonly used. However, pituitary-derived FSH requires multiple injections, contains luteinizing hormone (LH) and other proteins, and carries a risk of disease transmission. The development of recombinant FSH proteins through protein recombinant technology represents a significant research direction for addressing the aforementioned challenges. Over the past 30 years, extensive studies have been conducted on the development of recombinant bovine, ovine, or porcine FSH. Nevertheless, to date, no commercially recombinant FSH has been widely applied in the superovulation of cattle. This review provides an overview of the molecular design, selection of expression systems, and biological activity of recombinant bovine, ovine, or porcine FSH. This review also summarizes the results of utilizing recombinant FSH in superovulation protocols for cattle. The utilization of recombinant proteins as an alternative to traditionally extracted products in livestock production represents an irreversible trend. The livestock industry is highly sensitive to costs; therefore, it is essential to develop high-quality and cost-effective recombinant FSH products through appropriate molecular design and the use of suitable expression systems.

## Simple Summary

Reproductive technologies have become indispensable to modern livestock production. Embryo transfer technology is a key reproductive technique in cattle breeding, where superovulation is recognized as one of the most crucial steps in the procedure. Superovulation relies on the administration of exogenous hormones to stimulate follicular development and maturation in the ovaries, with pituitary-derived follicle-stimulating hormone being the most commonly used. However, pituitary-derived follicle-stimulating hormone requires multiple injections, contains luteinizing hormone and other proteins, and carries a risk of disease transmission. To address the aforementioned challenges, extensive studies have been conducted on the development of recombinant bovine, ovine, or porcine follicle-stimulating hormone. Nevertheless, to date, no commercially recombinant follicle-stimulating hormone has been widely applied in the superovulation of cattle. The focus of this review is to investigate the molecular design of recombinant bovine, ovine, or porcine follicle-stimulating hormone, the selection of expression systems for producing recombinant follicle-stimulating hormone, and the practical outcomes of utilizing recombinant follicle-stimulating hormone for the superovulation of cattle. The utilization of recombinant proteins in the reproductive processes of livestock represents an emerging trend in the field. The purpose of this review is to provide evidence-based references and recommendations for the development and application of recombinant follicle-stimulating hormone.

## 1. Introduction

Currently, with the continuous in-depth study of reproductive regulatory mechanisms, reproductive technologies have been widely applied in the production of livestock animals. Reproductive technologies have revolutionized modern livestock production by providing methods to enhance breeding efficiency, improve genetic traits, and increase overall productivity. Reproductive technologies have become indispensable to modern livestock production [1]. Embryo transfer technology is a key reproductive technique in cattle breeding, where superovulation is recognized as one of the most crucial steps in the procedure.

Superovulation is the technique that has been most broadly used and has the greatest impact on herd productivity [2]. It requires the artificial regulation of follicular growth and development within the ovaries of female mammals. During follicular development and maturation under physiological conditions, follicle-stimulating hormone (FSH) regulates a number of transcriptional and metabolic events that are essential for proliferation and differentiation in the ovaries of female mammals [3,4]. Therefore, FSH products are widely utilized in the reproductive management of livestock. Initially, FSH products were extracted from the pituitary glands of pigs and contained both FSH and luteinizing hormone (LH) [5]. However, several studies have revealed that high concentrations of LH can negatively impact the quality and production of bovine embryos [6,7]. Subsequently, highly purified porcine FSH, extracted from the pituitary after the removal of 80% of the LH, has been widely applied globally [7].

Currently, FSH products available on the market for veterinary applications are produced from porcine or ovine pituitary extracts [8,9]. These porcine or ovine pituitary extracts are commercially available and have been scientifically validated [10,11,12,13,14]. The application of FSH extracted from porcine pituitaries is the most widespread, possibly due to the easier availability of raw materials. Although pituitary-derived FSH (pFSH) has been utilized in the livestock industry for several decades, several issues remain to be addressed. The most critical aspect is that the half-life and clearance time of natural FSH are approximately 5 h and 10 h, respectively [15,16]. This indicates that up to eight doses need to be administered in a typical superovulation schedule. The high number of pFSH administrations not only requires a significant amount of labor to complete the superovulation schedule but also raises animal welfare concerns. Moreover, pFSH is always contaminated with LH and no related proteins from pituitary tissue [6,16,17]. Because of the analogous physical and chemical properties of FSH and LH, the complete removal of LH from pituitary extracts poses significant challenges. The presence of LH in extracted FSH products can lead to premature luteinization of follicles, which subsequently reduces the superovulatory response [7,18,19,20]. Therefore, greater purity of FSH is more likely to yield a better superovulatory response in cattle [6,21,22]. Furthermore, due to its porcine origin, porcine pFSH has the potential to induce an immune response when administered repeatedly to cattle, goats, and sheep [23]. The use of animal-derived products may also increase the risk of disease transmission [24]. Additionally, the costly process of FSH extraction increases the financial burden on the end user. The yield of FSH extracted from pituitary glands is limited by the availability of raw materials.

In response to the challenges associated with multiple administrations, significant effort has been directed toward simplifying treatments by reducing the number of pFSH administrations. The frequency of administration has been directly lowered from twice daily to once daily, yet the superovulatory response was inadequate [25]. However, once daily administration of FSH mixed with a 3.2% protein gelatin carrier vehicle improved the superovulatory response. [26]. Several studies have reported that a single administration of the recommended dose of pFSH mixed with polyvinylpyrrolidone [27,28] or aluminum hydroxide gel [29] also resulted in high superovulatory responses. However, the concern that superovulation with these substances may induce the production of antibodies against pFSH has prevented their adoption [30]. Hyaluronan, a biocompatible natural glycosaminoglycan, has been used as a diluent to support the sustained release of various drugs [31]. By dissolving pFSH in hyaluronan, several studies indicate that only one or two administrations are required to achieve a superovulatory response comparable to that obtained with twice daily administrations over four days [32,33,34]. The slow-release formulation strategy of pFSH achieved superovulatory responses that were comparable to those obtained with traditional protocols, indicating a potential to reduce labor demands, minimize stress on donors, and lower the risk of injuries to humans and cattle [35]. However, slow-release formulation protocols have not been widely adopted, possibly due to cost issues and the complexity of the formulation preparation. Therefore, it is necessary to explore alternative methods to address the challenges associated with the pituitary-derived products.

In human assisted reproductive technologies, FSH products, including urinary FSH (u-FSH) and recombinant FSH, have been widely used to promote the growth and maturation of ovarian follicles [36]. A single treatment of long-acting recombinant human FSH could effectively replace the daily treatments of short-acting urinary or recombinant FSH, thereby making assisted reproductive technology more patient-friendly [36,37,38,39]. However, for the past few decades, only short-acting pituitary-derived FSH has been used to stimulate follicular growth and development in cattle. By drawing on successful strategies from human pharmaceutical development, the application of recombinant protein technology to develop long-acting FSH may provide an effective alternative to pituitary-derived FSH for stimulating follicular development and maturation in cattle during superovulation.

With the integration of recombinant DNA technology and protein expression systems, target proteins can be effectively secreted using engineered cell cultures in vitro. These proteins are free from other impurities and exhibit biological activities and functions that closely match those of proteins typically secreted by the body. To produce target recombinant proteins, the process starts by constructing complete DNA sequences that express the protein and then proceeds with the development of a stable, genetically engineered cell line that can secrete the target protein. Therefore, the focus of this review is to investigate the molecular design of recombinant bovine, ovine, or porcine FSH, the selection of expression systems for producing recombinant FSH, and the practical outcomes of utilizing recombinant FSH for the superovulation of cattle.

## 2. Molecular Characteristics of Natural FSH

FSH is a pituitary secretory glycoprotein hormone and contains a generic α-subunit and a highly specific β-subunit that couple to each other via non-covalent interactions [40]. Both the α-subunit and β-subunit exhibit no biological activity when they are present alone; however, they regain this activity upon recombination [41]. Unlike small-molecule drugs, proteins are complex macromolecules arising from a combination of their large size and higher-order structures. The structurally complex conformations of proteins influence their specific biological activities [42]. The non-covalent interactions of the αβ heterodimer are essential for exhibiting the bioactivity specified by the identity of the β-subunit [41,43,44,45,46,47]. The tertiary structure of FSH is formed by the α-subunit and β-subunit linked by intramolecular disulfide bonds, which is crucial for its in vivo biological activity [48].

Both the α-subunit and β-subunit are modified by two sites of N-glycosylation, and their three-dimensional structure is characterized by three loops [49]. Glycosylation is essential for the in vivo biological function and bioactivity of FSH [50], which is not only a vital factor in determining its survival in the circulation [51] but also triggers the cellular signaling pathway through the activation of the FSH receptor [52,53]. Each glycan at one of the four N-glycosylation sites plays a crucial role. The oligosaccharides of the α-subunit are indispensable for α-subunit folding and stability [54,55], heterodimer stability [56], and FSH receptor binding and activation [52,57]. The two N-glycans of the β-subunit contribute significantly to metabolic clearance [51]. Moreover, terminal sialic acid residues are crucial for determining the heterogeneity of FSH isoforms and serve as a key structural component of glycoprotein hormones [58]. Therefore, the physicochemical and biochemical characterization of recombinant FSH is essential for assessing its quality, stability, and biological potency [59].

## 3. Molecular Design of Recombinant FSH Proteins

The molecular design of recombinant FSH currently includes four distinct variants. One strategy involves linking the α-subunit and β-subunit together through non-covalent interactions, emulating the natural molecular assembly processes. Another strategy involves the formation of a single-chain structure through the tandem fusion of the α-subunit and β-subunit. The other two strategies involve either linking the α-subunit and β-subunit through non-covalent interactions or forming a single-chain structure via tandem linkage following the fusion of exogenous amino acid sequences. The primary purpose of fusing exogenous amino acid sequences is to extend the half-life of recombinant FSH. Additionally, single-chain molecules have three main advantages. One advantage is that only a single plasmid is required to produce an active recombinant FSH, which simplifies the production process and reduces associated costs. Another advantage is that the two subunits are consistently expressed in equal amounts, thereby avoiding the unnecessary synthesis of excess free subunits. The third advantage is that the subunits maintain close proximity, which facilitates the formation of the bioactive three-dimensional structure of recombinant FSH [60].

Before 2000, the molecular design of recombinant FSH primarily involved the formation of a heterodimer through non-covalent linkage of the α-subunit and β-subunit [61,62,63,64,65,66]. Since 2000, several single-chain and long-acting molecules have been developed, expressed through various expression systems, and subjected to both in vitro and in vivo biological assays. A single-chain bovine FSH (sc-bFSH) was constructed by fusing the carboxyl end of the β-subunit to the amino end of the α-subunit via overlap PCR [67]. Fidler et al. reported that a biologically active single-chain ovine FSH (oFSHβα) was developed with the C-terminus of the β-subunit linked to the N-terminus of the α-subunit through a two-amino acid linker sequence [48].

Luo et al. reported that a recombinant HSA-pFSH fusion protein was developed by linking a truncated mature human serum albumin (HSA) fragment to the N-terminus of porcine FSHβ sequence through a flexible Gly-Gly-Gly-Gly-Ser linker [68]. A novel, hyperglycosylated, long-acting recombinant bovine FSH (LA-rbFSH) was developed by fusing the β-subunit with three copies of a highly O-glycosylated peptide derived from modified human granulocyte and macrophage colony-stimulating factor (mGMOP). The pharmacokinetic study results indicated that the elimination half-life of LA-rbFSH was 14 ± 2 h [69]. The α-subunit of these two molecules was linked to the modified β-subunit through non-covalent interactions.

**Figure 1:**
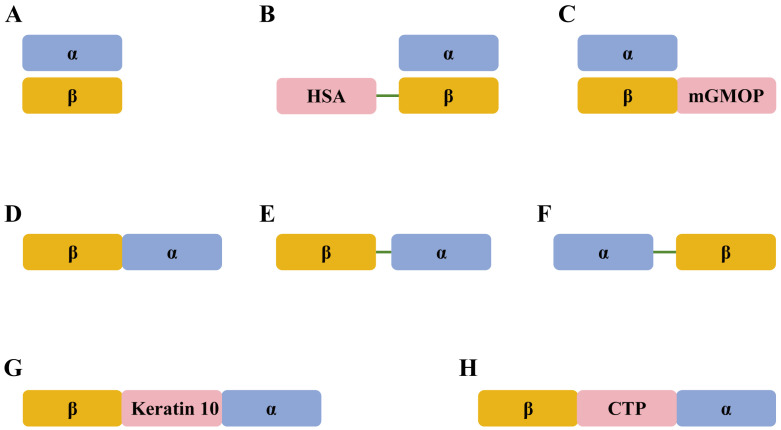
The structures of different variants of recombinant FSH. (**A**) The native form of FSH consists of an α-subunit and a β-subunit that are linked together by non-covalent interactions. (**B**) The α-subunit and β-subunit are linked by non-covalent interactions, with the N-terminus of the β-subunit connected to a truncated mature human serum albumin (HSA) fragment via a flexible Gly-Gly-Gly-Gly-Ser linker. (**C**) The α-subunit and β-subunit are linked by non-covalent interactions, with three copies of mGMOP highly O-glycosylated peptide fused to the C-terminus of the β-subunit. (**D**) A single-chain FSH is constructed by fusing the carboxyl end of the β-subunit to the amino end of the α-subunit via overlap PCR. (**E**) A single-chain FSH is developed with the C-terminus of the β-subunit linked to the N-terminus of the α-subunit through a two-amino acid linker sequence. (**F**) A single-chain FSH is engineered by linking the α-subunit and β-subunit via a flexible spacer peptide that includes two potential N-glycosylation sites. (**G**) A single-chain recombinant bovine FSH (bscFSH) is constructed by fusing the β-subunit and α-subunit of bovine FSH with a 33-amino acid spacer peptide, which was derived from human keratin 10, rich in glycine and serine, and featured two potential N-glycosylation sites integrated into its internal portion. (**H**) A single-chain FSH was constructed, consisting of the β-subunit and α-subunit linked by a carboxyl-terminal peptide (CTP) linker.

Gutiérrez-Reinoso et al. reported that a single-chain recombinant bovine FSH (bscrFSH) was engineered by linking the α-subunit and β-subunit via a flexible spacer peptide that included two potential N-glycosylation sites [70]. Cabeza et al. reported that a single-chain recombinant bovine FSH (bscFSH) was constructed by fusing the β-subunit and α-subunit of bovine FSH with a 33-amino acid spacer peptide. This spacer peptide, derived from human keratin 10, was rich in glycine and serine, and featured two potential N-glycosylation sites integrated into the internal portion of this peptide [2]. Abreu et al. described the creation of a single-chain β-CTP-α chimera of recombinant bovine FSH (scbFSH), consisting of the β-subunit and α-subunit linked by a carboxyl-terminal peptide (CTP) linker. [71]. The structures of different variants of recombinant FSH are summarized and presented in Figure 1.

Many single-chain FSH molecules have been developed and reported in both humans and animals [2,48,67,70,71,72,73,74,75,76]. Nevertheless, single-chain FSH has not been commercialized for clinical applications, possibly because of its potential to trigger an immune response, as these forms are not found in naturally occurring hormones [60,77]. To diminish immunogenicity, alternative sequences that are both flexible and non-immunogenic can be used to link the two subunits [78,79]. Another limitation to the development of single-chain gonadotropins is that they do not offer superior biological properties compared with their heterodimeric counterparts. In natural glycoprotein hormones, the two subunits linked by non-covalent interactions ensure the stability of the αβ heterodimer under physiological conditions [60]. Therefore, recombinant FSH, which is more congruent with the natural structure and linked by non-covalent interactions, may be the optimal molecular structure.

## 4. Expression Systems for Recombinant FSH Proteins Production

Currently, the most commonly used protein production systems are *E. coli*, yeast, insect cells, and mammalian cells. Each of these systems offers unique advantages and challenges, making them suitable for different applications and types of proteins. *E. coli* is the first choice for the production of prokaryotic target proteins. For simple eukaryotic target proteins that do not require post-translational modifications and that possess a limited amount of disulfide bonds, *E. coli* can be considered an expression host as well [80]. However, *E. coli* cannot perform most post-translational modifications, particularly glycosylation, and often fails in folding complex proteins containing multiple disulfide bonds, as well as large multi-domain assemblies and multi-subunit complexes [81]. Due to the characteristics of FSH, the recombinant FSH proteins have been expressed in various expression systems, including yeast, insect cells, plant cells, *Leishmania tarentolae*, and Chinese hamster ovary (CHO) cells.

Recombinant porcine FSH, whose α-subunit and β-subunit were coupled to each other by the non-covalent interactions, was produced in methylotrophic yeast. The yield of secreted recombinant FSH reached a maximum of 10 mg/L [63]. A biologically active single-chain ovine FSH (oFSHβα) was developed with the C-terminus of the β-subunit linked to the N-terminus of the α-subunit through a two-amino acid linker sequence and expressed in the methylotrophic yeast. The expression level of oFSHβα reached approximately 0.1 mg/L [48]. Recombinant HSA-pFSH fusion protein was developed by linking a truncated mature HSA fragment to the N-terminus of the porcine FSHβ sequence through a flexible linker. The recombinant HSA-pFSH was expressed in yeast and reached an expression level of 40.8 mg/L after purification [68]. Yeast cells are capable of both N-glycosylation and O-glycosylation; however, the patterns of glycosylation differ significantly from those observed in mammalian cells [82]. Specifically, yeast N-glycosylation is predominantly of the high-/hyper-mannose type, which can contribute to antigenicity [83].

Recombinant bovine FSH, linked by non-covalent interactions between the α-subunit and β-subunit, was secreted by baculovirus–insect cells. The yield, ranging from 1 to 5 mg/L, was achieved 70–90 h post infection [64]. Kato et al. reported that recombinant porcine FSH was synthesized by a baculovirus–insect cell system using two cDNAs encoding the FSH α-subunit and β-subunit. Recombinant porcine FSH was secreted into the culture medium at an approximate concentration of 1 mg/L [66]. The baculovirus–insect cell expression system is one of the most widely used systems for the production of heterologous protein, particularly membrane proteins, in both academia and industry [84,85]. However, N-glycans of recombinant proteins produced in insect cells are generally not processed into terminally sialylated complex-type structures; instead, they are modified into paucimannose or oligomannose structures [86]. This situation may affect the biological activity of recombinant FSH.

A single-chain bovine FSH (sc-bFSH) was expressed in a plant expression system, with an expression level of approximately 3% of the total soluble proteins [67]. Plant cells can perform post-translational modifications, including glycosylation and disulfide bridge formation [87,88]. However, the structure of mature N-glycan complexes differs to some extent between plants and mammals, as plant glycoproteins do not contain sialic acid [89]. A single-chain β-CTP-α chimera of recombinant bovine FSH was expressed via the *Leishmania tarentolae* expression system. After optimization of the culture conditions and purification processes, the average protein expression level of scbFSH reached 2.95 ± 0.14 mg/L [71]. *Leishmania tarentolae* has emerged as a cost-effective alternative expression host that can grow in high densities in vitro without requiring animal serum or CO_2_ [90]. Although the glycosylation in *Leishmania tarentolae* is very similar to mammalian-type N-glycosylation, it is devoid of terminal sialic acid [91].

Chinese hamster ovary (CHO) cells were cotransfected with the α-subunit and a hybrid β-subunit, in which the 5′ non-coding and signal peptide-coding sequences of the β-subunit cDNA were replaced with those derived from an ovine growth hormone (oGH) cDNA. This modification resulted in the highest protein expression levels, reaching 0.10 to 0.16 µg per 10^6^ cells per day [61]. Recombinant single-chain bovine FSH, constructed by fusing the β-subunit and α-subunit of bovine FSH with a 33-amino acid spacer peptide, was produced in CHO cell cultures, achieving an expression level of approximately 30 µg per 10^6^ cells per day [2]. The long-acting recombinant bovine FSH (LA-rbFSH) and a non-modified version (rbFSH) were produced in suspension CHO cell cultures. The productivities of the recombinant cell lines were 0.2 and 1.3 µg per 10^6^ cells per day for rbFSH and LA-rbFSH, respectively [69]. CHO cells are often preferred for the production of complex biopharmaceutical proteins due to their ability to replicate protein folding and post-translational modifications [92,93].

Different expression systems possess various advantages and disadvantages. A comparison of different expression systems is presented in Table 1. The prokaryotic expression system is the most commonly used expression system, characterized by its high expression efficiency, low cost, and relatively simple operational procedures. However, it lacks the ability to perform post-translational modifications on proteins, making it suitable mainly for structurally simple proteins. Additionally, there is a significant concern regarding the need to strictly control endotoxin levels when using this system. Yeast cells provide several advantages, including cost-effectiveness and ease of purification. Additionally, they are capable of performing some post-translational modifications, making them suitable for producing functional eukaryotic proteins. However, the glycosylation of proteins expressed in yeast differs from that of mammalian proteins. This difference in glycosylation patterns can not only affect the biological functions of the expressed proteins but may also induce immune responses when these proteins are administered to mammalian animals. Insect cell expression systems are characterized by high expression efficiency and are well suited for the production of complex proteins. However, although the glycosylation patterns of proteins expressed in insect cells resemble those of mammalian proteins, notable differences persist that may influence their biological activity. Additionally, insect cell expression systems tend to incur higher costs, involve more complex operational procedures, and necessitate longer production cycles compared to *E. coli* and yeast. Mammalian cell systems are ideally suited for the production of complex functional proteins, as they possess the ability to perform complex post-translational modifications. As a result, these systems have become the most widely employed expression platforms in the pharmaceutical industry. The glycosylation patterns of proteins expressed in mammalian cells closely resemble those of native proteins. However, mammalian cell expression systems are associated with higher costs, complex operational procedures, and longer production cycles. Therefore, for the production of recombinant FSH characterized by a high degree of glycosylation and a relatively complex structure, mammalian expression systems may be the optimal choice.

## 5. Biological Activity of Recombinant FSH Proteins

The biological activity of recombinant FSH proteins is determined by molecular design as well as the choice of the protein expression system. The correct tertiary structure of recombinant FSH is formed by the α-subunit and β-subunit linked by intramolecular disulfide bonds, which is crucial for the structural and physiological functions of recombinant FSH [40,94]. Moreover, the N-glycosylation and terminal sialic acid residues of recombinant FSH are essential for its biological activity [48,58]. Early studies assessing biological activity primarily relied on in vitro experiments comparing the activity of recombinant FSH with that of extracted FSH. The in vivo bioactivity of recombinant FSH is assessed using the Steelman–Pohley bioassay. This method evaluates the biological activity by comparing the increase in ovarian weight in immature female rats treated with the test FSH preparation against that observed with the international reference standard of FSH [95].

Inaba et al. reported that recombinant porcine FSH (rpFSH) linked by non-covalent interactions between the α-subunit and β-subunit was produced from baculovirus–insect cells. The calculated in vitro FSH activity in terms of purified pituitary FSH was 1.11 units/mg for rpFSH in the Sertoli cell aromatase bioassay. Furthermore, rpFSH alone could promote the development of follicles, and the subsequent treatment with hCG could trigger ovulation in hypophysectomized mice [62]. Another study reported that recombinant bovine FSH (rbFSH), linked by non-covalent interactions between its α-subunit and β-subunit, was secreted by baculovirus–insect cells. Recombinant bovine FSH has demonstrated high potency in various in vitro bioassays, with biological activities ranging from 6 to 23 IU/mL. However, the lack of binding of rbFSH to wheat germ agglutinin suggested that the glycosidic side chains may not contain terminal sialic acid [64].

In 2001, a recombinant single-chain bovine FSH (sc-bFSH) was expressed in a plant expression system. Two types of the plant paucimannosidic glycan, truncated forms of complex-type N-glycans, were found in purified sc-bFSH via mass spectrometric analysis. The promotion of cAMP production in CHO cells expressing porcine FSH receptor confirmed the native-like structure and bioactivity of sc-bFSH in vitro. The calculated in vitro FSH activity in terms of pituitary bFSH was 850 IU/mg for sc-bFSH. Although sc-bFSH exhibited effective in vivo biological activity in superovulation of mice, its activity was much lower than that of the pregnant mare serum gonadotropin (PMSG) treatment group [67].

A single-chain β-CTP-α chimera of recombinant bovine FSH (scbFSH) was expressed via the *Leishmania tarentolae* expression system. The in vivo bioactivity of scbFSH was evaluated through the measurement of ovarian weight gain in rats following subcutaneous administration. The calculated specific activities of scbFSH ranged from 44.4 to 56.3 IU/mg [71].

Villarraza et al. reported that a non-modified version of recombinant bovine FSH (rbFSH) and a long-acting recombinant bovine FSH (LA-rbFSH) were produced in suspension cultures of CHO cell. LA-rbFSH was developed by incorporating three copies of a highly O-glycosylated peptide, which is derived from modified glycoprotein oligomers (mGMOP), specifically into the C-terminal region of the β-subunit. LA-rbFSH presented a greater degree of glycosylation and sialic acid content than those of rbFSH. The efficacy of the recombinant FSH was evaluated by measuring the increase in ovarian weight in rats. The in vivo bioactivity of rbFSH and LA-rbFSH was 4200 and 10,000 IU/mg, respectively [69].

Mammalian cells predominantly produce complex-type N-glycans, characterized by glycan branches that are modified with N-acetylglucosamine, galactose, fucose, and sialic acid [96,97]. Mammalian cell lines used for protein production are typically derived from human embryonic kidney 293 (HEK293) cells or CHO cells. HEK293 cells are commonly employed in research applications due to their ease of transfection, while CHO cells are often preferred for the production of biopharmaceutical proteins. The commercialized recombinant human short-acting (follitropin alfa, GONAL-f) and long-acting FSH (FSH-CTP, corifollitropin alfa, Elonva) expression systems are both based on CHO cells. According to reports on the activity of recombinant bovine, ovine, or porcine FSH, the activity of recombinant FSH expressed in CHO cells is the highest, reaching 10,000 IU/mg. Therefore, mammalian cells, particularly CHO cells, may represent the optimal expression system for recombinant FSH protein. A summary of the molecular design and expression systems of recombinant bovine, ovine, or porcine FSH is presented in Table 2.

## 6. Application of Recombinant FSH in the Superovulation of Cattle

In the past 30 years, recombinant bovine, ovine, and human FSH proteins have been studied for their application in superovulation in cattle. Based on the molecular characteristics of recombinant FSH, superovulation in cattle was induced using three different administration protocols: twice daily over four days, once daily over four days, and a single administration. The results of the superovulation experiments conducted on cattle are summarized in Table 3.

In 1996, the earliest research on the application of recombinant bovine FSH for inducing superovulation in non-lactating crossbred beef cows was conducted. The recombinant FSH was developed by Granada Biosciences, Inc. A total of 24 mg of recombinant FSH was administered to each cow in equal doses twice daily for three days, beginning on day 8 after the insertion of progesterone-releasing intravaginal devices (PRIDs). The number of transferable embryos was 2.4 ± 0.9 in the 0.5 PRID-treated group and 3.0 ± 0.9 in the 2.0 PRIDs-treated group [98]. Another study compared the effects of three hormones on superovulation in Holstein cows: Folltropin (pituitary-derived porcine FSH), Cinnal-f (recombinant human FSH), and menotropins (hMG). Three gonadotropin products were given twice daily in decreasing doses over 4 days. No significant differences were found in the number of transferable embryos among donors treated with Cinnal-f (5.1 ± 0.86), menotropins (6.3 ± 1.74), or Folltropin (5.1 ± 1.16) [99].

A single-chain recombinant bovine FSH (bscrFSH) was engineered by linking the α-subunit and β-subunit with a flexible spacer peptide, which included two potential N-glycosylation sites. Preliminary trials revealed that bscrFSH had a half-life in the circulation that exceeded 48 h. Unlike pituitary-derived porcine FSH, which was treated twice daily over four days, bscrFSH was treated once daily for four consecutive days in primiparous red Angus cows. The numbers of viable embryos in the bscrFSH group and the extracted FSH group were 8.65 ± 0.67 and 6.32 ± 0.56, respectively [70]. The effects of bscrFSH and extracted FSH on ovarian superovulatory responses have also been tested in Holstein heifers inseminated with unsorted or sex-sorted semen. In comparison with the extracted FSH treatment group, the bscrFSH treatment group presented greater numbers of transferable embryos with both unsorted (7.60 ± 1.27 vs. 4.12 ± 0.38) and sex-sorted semen (4.10 ± 0.88 vs. 1.89 ± 1.01) [100]. Another single-chain recombinant bovine FSH (bscFSH) variant, in which the β-subunit and α-subunit of bovine FSH were fused by a 33-amino acid spacer peptide derived from human keratin 10, was designed and expressed in CHO cells. In superovulation studies with cattle, the administration of bscrFSH at a dose of approximately 0.5 μg/kg body weight, following a decreasing dosage schedule over four doses with 24 h intervals, led to an average of eight–twelve transferable embryos per animal [2].

In 2014, one study compared the effects of two long-acting recombinant bovine FSH (rbFSH; types A and B) and pituitary-derived porcine FSH (Folltropin) on superovulation in Holstein heifers. Two types of long-acting recombinant bovine FSH were developed by CEVA Animal Health. The animals received a total of eight decreasing doses of Folltropin or a single injection of two types of long-acting recombinant bovine FSH. There were no statistically significant differences in the number of good-quality embryos among the treatment groups: 50 μg recombinant B-type FSH (7.6 ± 2.4), 100 μg recombinant A-type FSH (4.3 ± 1.5), and Folltropin (6.5 ± 1.7) [101]. In 2020, a recombinant long-acting ovine FSH (roFSH), which included additional N-glycosylation sites and had a long half-life in the circulation after intramuscular administration, was developed (AgResearch New Zealand Ltd., confidential report). An average of 6.1 good-quality embryos were harvested following treatment with a single administration of roFSH [102]. Commercial long-acting human recombinant FSH (rhFSH) has been used to evaluate its effectiveness in the superovulatory response of cattle. The rhFSH is composed of an FSH β-subunit linked to the C-terminal peptide (CTP) of the β-subunit of human chorionic gonadotropin and is expressed in CHO cells [103]. A single subcutaneous injection of 25 μg of rhFSH was administered to induce superovulation in Girolando and Nelore cows and heifers. The number of viable embryos was 5.8 ± 1.6 and 6.4 ± 1.0, respectively [104].

**Table 3:** In vivo production of bovine embryos by different recombinant FSH.

Year	Species	Molecular Design	Expression System	Viable Embryos	Reference
1996	bovine	NR	NR	2.4 ± 0.9 and 3.0 ± 0.9	[98]
2014	bovine	NR	NR	4.3 ± 1.5 (type A)	[101]
2014	bovine	NR	NR	7.6 ± 2.4 (type B)	[101]
2020	ovine	NR	NR	averages of 6.1	[102]
2022	human	NR	NR	5.1 ± 0.86	[99]
2022	bovine	α and β linked by a flexible spacer peptide	CHO	8.65 ± 0.67	[70]
2023	bovine	α and β linked by a flexible spacer peptide	CHO	unsorted (7.60 ± 1.27) and sex-sorted semen (4.10 ± 0.88)	[100]
2024	human	α and β-CTP non-covalently linked	CHO	5.8 ± 1.6 and 6.4 ± 1.0	[104]
2024	bovine	β and α linked by a 33-amino acid spacer peptide	CHO	average of 8–12	[2]

NR, not reported; CHO, Chinese hamster ovary; CTP, carboxyl-terminal peptide.

## 7. Discussion

Recombinant FSH protein applications in human assisted reproduction have been developed and validated over several decades. Commercially available long-acting FSH and short-acting FSH have been widely applied in human clinical practice [103,105,106]. The use of recombinant FSH proteins as an alternative to extracted FSH products in livestock production represents an irreversible trend. Moreover, the livestock industry is highly cost-sensitive and requires more commercial products. Additionally, FSH is a heterodimeric protein composed of α-subunit and β-subunit linked by non-covalent interactions. The N-glycosylation and terminal sialic acid residues are critical for the biological function and activity of FSH [40,50,58]. Consequently, the molecular design of recombinant FSH must ensure that the α-subunit and β-subunit can properly assemble to form a heterodimer. Given the importance of N-glycosylation and sialic acid residues in FSH, an appropriate protein expression system must be selected.

Currently, the widely used pituitary-extracted FSH requires eight injections over four days, and the development of recombinant long-acting FSH with an appropriate half-life could be an effective approach to reduce the number of administrations. Although equine chorionic gonadotropin (eCG), also known as pregnant mare serum gonadotropin (PMSG), was initially used for superovulation in cattle, its excessively long half-life led to abnormal endocrine disorders, follicular ovulation, and a decline in oocyte quality [107]. The intravenous administration of antibodies against PMSG could ameliorate this situation at the time of the first insemination [108,109]. Therefore, the half-life of recombinant long-acting FSH should not exceed that of PMSG, which has a half-life of more than 40 h in cattle [110,111,112].

Although numerous recombinant bovine, ovine, porcine, and human FSH proteins have been reported for use in cattle production applications, the FSH predominantly utilized in superovulation treatments is the FSH extracted from porcine pituitary glands. According to the detection results from the UniProt Knowledgebase, the homology percentages for the FSHα-subunit are as follows: 74.14% between bovine and human, 96.67% between bovine and pig, and 99.17% between bovine and sheep. For the FSHβ-subunit, the homology percentages are 88.37% between bovine and human and 93.80% between bovine and both pig and sheep. The α-subunit in human FSH consists of 116 amino acids, while the α-subunits in pig, bovine, and sheep each comprise 120 amino acids. Additionally, the β-subunits in all four species are composed of 129 amino acids. Both human FSH and FSH from pig, bovine, and sheep possess two N-glycosylation sites and five disulfide bonds in their α-subunits. However, due to the shorter amino acid sequence of the human α-subunit compared to the other three species, the positions of the glycosylation sites and disulfide bonds differ from those in pig, bovine, and sheep FSH. In contrast, the N-glycosylation sites and disulfide bonds in pig, bovine, and sheep are consistently located at the same positions. Notably, the α-subunit in bovine FSH has an O-glycosylation site at the 67th amino acid position. Regarding the β-subunit, all four species contain two N-glycosylation sites and six disulfide bonds, with the positions of the glycosylation sites and disulfide bonds being consistent across all four species. FSH is relatively conserved among different species. Due to the high similarity of FSH among pigs, bovine, and sheep, FSH extracted from the pituitary glands of pigs or sheep can be effectively utilized in superovulation protocols for cattle. Furthermore, recombinant porcine, bovine, or ovine FSH has been developed to improve reproductive performance in cattle.

According to current research reports, the optimal expression system for recombinant FSH protein at this stage is the CHO cell expression system, which maximally preserves the biological activity of FSH. The simplest molecular design approach is to develop wild-type FSH as a substitute for extraction products, followed by the development of long-acting molecules to reduce the frequency of administration. However, the design of long-acting molecules should not adversely affect the assembly and activity of FSH. Most importantly, it is essential to select high-yield cell lines that express highly active recombinant proteins, optimize cell culture processes, and refine protein purification methods to reduce the production costs of recombinant proteins. This, in turn, will lower end-use costs, making it feasible to promote the application of recombinant FSH in the reproductive production of livestock. We believe that, in the near future, recombinant FSH proteins will be applied in livestock production.

## 8. Conclusions

The utilization of recombinant proteins in the reproductive processes of livestock represents an emerging trend in the field. However, there are currently no commercialized recombinant products widely applied in actual production. According to the existing research reports, developing recombinant long-acting FSH with a suitable half-life, correct glycosylation, and high biological activity via an appropriate protein expression system is likely to be a major research and development direction in the future. Enhancing protein expression and reducing production costs by optimizing cell-culture conditions for recombinant proteins are also vital for application in animal husbandry production. Following the successful development of cost-effective recombinant FSH proteins with high biological activity, these proteins are expected to provide high-quality and affordable products for livestock production due to their advantages of high activity, high purity, and the absence of limitations on raw materials.

## Figures and Tables

**Table 1:** Comparison of different expression systems.

Expression System	Advantages	Disadvantages
*E. coli*	High expression efficiency, low cost, and simple operational procedures	Lack of post-translational modifications and control endotoxin levels
Yeast	The cost-effectiveness and ease of purification	The glycosylation differs from that of mammalian proteins
Insect cell	The high expression efficiency and production of relatively complex proteins	The glycosylation differs from that of mammalian proteins, higher costs, more complex operational procedures, and longer production cycles
Mammalian cell	Complex post-translational modifications	Higher costs, complex operational procedures, and longer production cycles

**Table 2:** The molecular design and expression system of recombinant FSH.

Species	Molecular Design	Expression System	Expression Level	In Vitro Bioassay	In Vivo Bioassay	Half-Life	Reference
ovine	α and β non-covalently linked	CHO cell	0.10–0.16 pg/cell/day	Activity in the testis radioreceptor assay and in vitro Sertoli cell bioassay	NR	NR	[61]
porcine	α and β non-covalently linked	Insect cell	NR	Estradiol production in Sertoli cell assay; in vitro bioactivity of 1.11 units/mg	Mouse uterine weight assay and ovulation; ovarian and uterine weights in rat	NR	[62]
porcine	α and β non-covalently linked	Yeast	10 mg/L	cAMP production in CHO cells; progesterone production in Y_1_ cells	NR	NR	[63]
bovine	α and β non-covalently linked	Insect cell	1–5 mg/L	Y_1_ cell-rounding assay; Y_1_ cell cAMP assay; cAMP in Sertoli cell assay; GVBD of bovine oocytes; in vitro bioactivity of 6–23 IU/ml	NR	NR	[64]
porcine	α and β non-covalently linked	Insect cell	NR	Stimulation of ovarian tPA enzyme activity	Induces ovulation in rats	NR	[65]
porcine	α and β non-covalently linked	Insect cell	1 mg/L	Progesterone in granulosa cells; GVBD of porcine oocytes	NR	NR	[66]
bovine	β and α fused via overlap PCR	Plant cell	3% of the total soluble proteins	cAMP production in CHO cells; in vitro bioactivity of 850 IU/mg	Superovulatory of mice	NR	[67]
ovine	β and α linked by a two amino acid linker	Yeast	0.1 mg/L	cAMP production in CHO cells	NR	NR	[48]
porcine	α and HSA-β non-covalently linked	Yeast	40.8 mg/L	cAMP production in HEK293 cells	NR	NR	[68]
bovine	α and β linked by a flexible spacer peptide	CHO cell	NR	nr	Superovulation and pharmacokinetics in cattle	circulating half-life higher than 48 h	[70]
bovine	β and α linked by a 33-amino acid spacer peptide	CHO cell	30 pg/cell/day	NR	Superovulation in cattle	NR	[2]
bovine	β and α linked by CTP linker	*Leishmania tarentolae*	2.95 ± 0.14 mg/L	NR	In vivo bioactivity of 56.3 IU/mg in SP bioassay	NR	[71]
bovine	α and β non-covalently linked	CHO cell	0.2 pg/cell/day	NR	Pharmacokinetics in rat; in vivo bioactivity of 4200 IU/mg in SP bioassay	12.80 ± 0.03 h	[69]
bovine	α and β-mGMOP non-covalently linked	CHO cell	1.3 pg/cell/day	NR	Pharmacokinetics in rat; in vivo bioactivity of 10,000 IU/mg in SP bioassay	14 ± 2 h	[69]

CHO, Chinese hamster ovary; NR, not reported; GVBD, germinal vesicle breakdown; tPA, tissue-type plasminogen activator; cAMP, cyclic adenosine monophosphate; HSA, human serum albumin; CTP, carboxyl-terminal peptide; SP, Steelman–Pohley assay; mGMOP, modified human granulocyte and macrophage colony-stimulating factor.

## Data Availability

Not applicable. The data presented in this manuscript were extracted from published literature.
